# NOMAD: metagenomic characterisation of the viral pathogen composition in outbreaks of non-malaria acute febrile illness cases

**DOI:** 10.12688/openresafrica.13406.1

**Published:** 2022-06-09

**Authors:** Benard W. Kulohoma, Ibrahim Ng'eno

**Affiliations:** 1 Centre for Biotechnology and Bioinformatics, University of Nairobi, Nairobi, Kenya; 2 International Centre for Insect Physiology and Ecology, Nairobi, Kenya

**Keywords:** Metagenomics, viral pathogens, non-malaria acute febrile illness, bioinformatics, detection

## Abstract

The clinical importance of non-malaria febrile acute illness (NM-AFI) in patients with a negative parasitological test has become apparent, with the progressive reduction in malaria transmission in endemic regions. Bacterial pathogens, for example *Streptococcus pneumoniae* and *Haemophilus influenzae*, which contribute disproportionally to febrile illness, are now preventable by vaccines. However, there are no vaccines, and little is known about viral NM-AFI prevalence, proliferation, virulence, and transmission chains between hosts. Although the predominant viral causes of NM-AFI are established, it is unclear if there are other NM-AFI associated emerging infectious viral pathogens that previously remained undetectable by conventional diagnostic strategies, for example severe acute respiratory syndrome coronavirus 2 (SARS-CoV-2). Presumptive broad-spectrum antibiotic prescriptions to aparasitaemic patients not only drive drug resistance, but also lead to poor treatment outcomes. We hypothesized that insights on NM-AFI etiology, and consequently case management, could be improved by exploiting viral sequence diversity to identify viral pathogens present within metagenomics samples. We exploited simulated and existing infectious disease (Ebola, hepatitis C, chikungunya, and mosquito-borne arboviruses) metagenomic datasets to determine the composition of viral pathogens present, by implementing profile Hidden Markov Models derived from Swiss-Prot viral reference sequences for accurate pathogen detection and classification. Our analysis identified a combination of sequences from multiple viral etiological agents within the same disease sample. This approach provides a granular perspective of multiple viral etiological agents present within a single intra-host disease episode. It highlights prevalent viral strains that can subsequently be routinely detected using directed diagnostic tests to improve disease surveillance in endemic regions.

## Introduction

Non-malaria febrile acute illness (NM-AFI) non-specifically refers to any illness presenting with fever and general malaise, except malaria, without a focal point of infection ^1, 2^. There is a paucity of information on NM-AFI prevalence, vectors and transmission, virulence, and associated socio-economic impacts ^3^. In Africa, previous efforts to understand the etiology of NM-AFI have largely been obscured by significant attention towards management of the malaria burden ^3^. Increasing use of rapid diagnostic tests (RDTs) of suspected malaria cases prior to treatment, and the decreasing trend of malaria transmission in endemic regions, now highlights the importance of NM-AFI in patients with a negative malaria test ^4^. Insensitive, slow, and unspecific diagnostic tests for NM-AFI are not beneficial in guiding pragmatic life-saving treatment. Divergent case definitions, inclusion and exclusion criteria during study enrolment, in some cases absence of control groups, focus on specific pathogens, and lack of standardized methodology have made it challenging to compare NM-AFI reports, trends and corresponding etiology between endemic regions ^1^.

Respiratory tract infection caused by bacterial and viral pathogens is a leading cause of NM-AFI in African children ^4– 7^. Previous NM-AFI etiology studies have focused on bacterial sepsis and highly prevalent viruses, for example dengue, chikungunya and human influenza virus type 2. They fail to highlight the possible contribution of other viral pathogens present during outbreaks, which are undetected by conventional laboratory assays. Distinct pathogen detection strategies with differing sensitivities used in these studies also complicate comparisons of etiological findings ^1^. In the absence of robust etiology information, presumptive broad-spectrum antibiotic treatment of patients who return a negative malaria test still drives drug resistance ^4, 8, 9^. Bacterial pathogens, *Streptococcus pneumoniae* and *Haemophilus influenzae,* which contribute disproportionally to febrile illness, are now preventable by conjugate vaccines ^10^. Although influenza type A is preventable by vaccination, most common respiratory viruses causing NM-AFI, including human parainfluenza virus 3 and respiratory syncytial virus, do not have vaccines and remain largely unidentified by clinical diagnostic tests.

Metagenomic datasets present ideal scenarios to examine the composition of viral communities. MinION nanopore sequencing is inexpensive to set-up compared to other technologies, portable, and does not require dedicated technician support or maintenance compared to other sequencing technologies, is gaining popularity as a diagnostic tool for identification of clinical pathogens in resource poor settings, such as those in disease endemic regions in sub-Saharan Africa ^11^. This sequencing technique enables real-time metagenomic detection of clinically relevant viral pathogens, and has previously been used in the surveillance of Lassa, Zika and Ebola virus ^12– 14^. Algorithms that robustly detect viral sequences present at low frequencies in these metagenomic datasets are crucial for determining the composition of pathogens that could be associated with disease. Although some tools, for example BLAST, are able to identify putative viral sequences, they are not able to robustly provide detailed pathogen classification profiles up to strain level, especially from very small stretches of sequence data ^15, 16^. We hypothesised that insights on NM-AFI etiology, and consequently case management, could be improved by exploiting viral sequence diversity to identify viral pathogens present within metagenomics samples. Metagenomic analysis strategies enable interrogation of the composition of viruses present without *a priori* knowledge of their identities, and reduce dependence on culture-based methods ^17^. Approximately 40% and 60% of the causative pathogens in gastroenteritis and encephalitis cases cannot be resolved using conventional laboratory assays ^18^. Presuming pathogen presence, remaining undetectable might be a consequence of exclusion in the diagnostic algorithm or a result of novel pathogen emergence, for example the Middle East respiratory syndrome coronavirus (MERS-CoV) and severe acute respiratory syndrome coronavirus 2 (SARS-CoV-2) that are of zoonotic origin ^19, 20^. We leverage metagenomic sequence data previously generated to establish the composition of viral pathogens that cause NM-AFI. We developed a robust bioinformatics detection pipeline, NOMAD ( **N**on- **M**alaria **A**cute febrile illness **D**etector) ^21^, capable of detecting multiple viral pathogens, and examined its ability to establish their abundance and accurately classify them in to respective taxa. NOMAD provides a granular perspective of the viral population diversity by exploiting difference in genetic sequence heterogeneity. 

## Methods

### Viral metagenomic datasets and virus detection

Metagenomic datasets consisting of viral sequences were retrieved from GenBank’s Sequence Read Archive (SRA) (19 ^th^ October 2020), using the SRA toolkit (version 2.9.0) ^22^. The metagenomic datasets were generated using MinION nanopore sequencing to detect chikungunya virus [SRA:SRP057410], Ebola virus [SRA:SRP057409 and SRA: SRX1023619], hepatitis C virus [SRA: SRP057418], and arboviruses in mosquito microbiomes [SRA:SRP121056 (runs SRR6205713, SRR6205714, SRR6205715 and SRR6205716)] ^11, 23^. Plasma samples containing chikungunya virus were collected from donors from Puerto Rico during the peak weeks of 2014 ^11^. Blood samples containing Ebola virus were collected from patients with suspected hemorrhagic fever during a 2014 outbreak in the Democratic Republic of Congo ^11^. The hepatitis C virus sample was obtained from an archived patient sample by the University of California, San Francisco ^11^. Consent was sought prior to sample collection, and ethical approval to conduct the studies at respective institutional boards. Since there is no gold standard for metagenomics analysis, simulated datasets are effective in evaluating data analysis performance.

NOMAD’s ability to classify sequences into taxa and estimate viral species abundance was examined using custom metagenomic datasets generated using iMESS ^24^. NOMAD’s performance was then compared to previous analysis by Bracken (Bayesian Re-estimation of Abundance after Classification with KrakEN), a popular tool used for such analyses, on the same metagenomic datasets ^25^. NOMAD’s advantage over bracken is the ability to detect subtle distinctions between strains from the same species, and not just the highest common ancestor if variants are detected. These metagenomic datasets consisted of MinION nanopore sequence reads generated from concentrations of 1 × 10 ^5^ copies/mL for the hepatitis C virus, and 1.0 × 10 ^7^ to 10 ^8^ copies/mL for Ebola and chikungunya respectively ^11^.

### Implementation

Profile Hidden Markov Models (HMMs) provide a probabilistic framework for identifying distant homologs and are well suited for detecting viral sequences ^16, 26^. Manually curated (n=563,630, as of 26 ^th^ October 2020) and computer-annotated (n=1,200,724, as of 26 ^th^ October 2020) viral protein sequences were retrieved from the Swiss-Prot and TrEMBL protein databases respectively ^27^, and used to construct profile HMMs. Rapid implementation of accelerated profile HMM searches was performed using HMMER (version 3.3. 2) ^28^, which achieves a similar speed as BLAST. Briefly, each sequence and its duplicate were aligned using MUSCLE ^29^. We used HMMER to estimate individual profiles from each of these alignments, and then concatenated the profiles. The nucleotide query sequences in FASTA format were translated in all six reading frames using transeq in the EMBOSS software suite ^30^, prior to use as input data. The resulting profile HMM was implemented using hmmscan from HMMER to align the input protein query sequences to the profile. An e-value threshold of < 1e-3, a measure of the expected number of false positives given the size of the database, was applied to retain statistically significant hits. Abundance determined by retention of duplicate reads, which was considered as the relative abundance of reads from each taxa ^31^. Customized shell scripts were used to perform annotation and display the final summary statistics.

### Operation

The average analysis runtime for ~50,000 metagenomics reads was 60 minutes. This analysis was conducted on our distributed HPC cluster, with an Ubuntu 20.04.3 LTS operating system, with each node being an Intel(R) Xeon(R) Silver 4214 CPU @ 2.20GHz with 12 cores.

## Results

**Figure 1.**
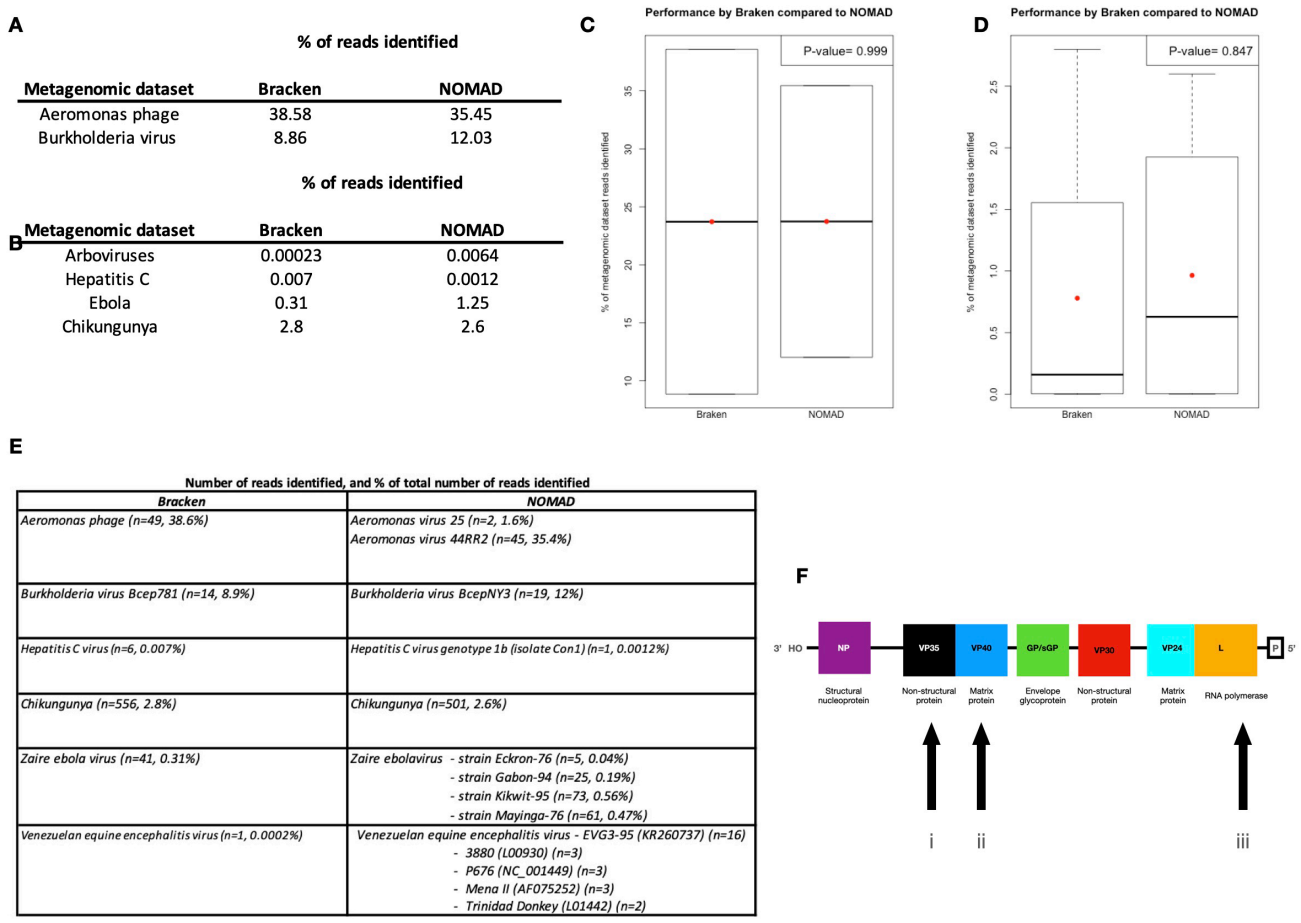
Comparison of performance by Bracken and NOMAD. Percentage of ( **A**) simulated and ( **B**) MinION nanopore metagenomic dataset reads identified by NOMAD and Bracken. Box plots comparing performance by NOMAD and Bracken across the ( **C**) simulated and ( **D**) MinION nanopore metagenomic datasets. The red dot shows the mean and the black line the median. MinION nanopore metagenomic dataset. ( **E**) The number of reads and percentage of the total dataset identified for each strain/lineage detected by Bracken and NOMAD. ( **F**) The genes present in Zaire Ebola virus showing the MinION nanopore metagenomic dataset three genes used to confirm the presence of multiple strains in the metagenomic dataset.

We first examined NOMAD’s ability to detect viral sequences using two simulated metagenomic datasets generated by iMESS composed of *Aeromonas* phage and *Burkholderia* virus sequences respectively ( *Extended data* ^32^). NOMAD’s ability to identify viral species sequences was not significantly different to performance by Bracken on the same datasets, Welch two sample t-test *p-value* = 0.9993, 95% CI (-85.8 - 85.8) ( Figure 1A and C). Analysis of the first simulated metagenomic dataset using Bracken identified *Aeromonas* phage reads present (n=49, 38.6%). NOMAD was not only able to identify *Aeromonas* phage reads, but also to specifically distinguish between the GC-poor phages *Aeromonas virus 25* (n=2, 1.6%) and *Aeromonas virus 44RR2* (n=45, 35.4%) reads present in this dataset. In the second simulated metagenomic dataset, Bracken identified *Burkholderia virus Bcep781* reads present (n=14, 8.9%), while NOMAD identified *Burkholderia virus BcepNY3*, a new member of the *Burkholderia phage Bcep781* family (n=19, 12%).

Comparisons of NOMAD’s performance to that by Bracken in analysis across the metagenomic datasets generated by MinION nanopore also showed no significant difference, Welch two sample t-test *p-value =* 0.8469, 95% CI (-2.4 - 2.1) ( Figure 1B and D). In the first dataset, Bracken detected a single read in the analysis of female *Culex cedecei* mosquito microbiome datasets generated to detect arboviruses. NOMAD detected multiple *Venezuelan equine encephalitis virus* (VEEV) variants, which include: EVG3-95 (KR260737) (n=16); 3880 (L00930) (n=3); P676 (NC_001449) (n=3); Mena II (AF075252) (n=3); and Trinidad Donkey (L01442) (n=2), present in the database of 144 VEEV genomes ( Figure 1E). In a separate analysis NOMAD was able to detect a single hepatitis C virus genotype 1b (isolate Con1) read ( Figure 1E), which is distinct from subtypes 1a, while Bracken detected only six reads of hepatitis C virus genotype 1. In metagenomic analysis of Caribbean strain of chikungunya virus isolated from a patient at the peak of the 2014 epidemic in Puerto Rico (strain PR-S6) ^11, 33^, Bracken detected 556 (n=2.8%) reads, while NOMAD identified 501 (2.6%) ( Figure 1E). Analysis of the metagenomic datasets from the August 2014 DRC Ebola virus with Bracken identified 41 (0.31%) reads, while NOMAD detected 164 (1.25%) ( Figure 1E). Results from the NOMAD analysis suggests the presence of multiple Ebola virus variants, raising the possibility that these were artefacts due to sequencing errors or that multiple linages of Zaire ebolavirus are present within the same intra-host disease episode, namely: Eckron-76, Gabon-94, Kikwit-95 and Mayinga-76. We examined whether these detected strain reads were from multiple loci or from a single locus. Multiple variants were identifiable at three loci: non-structural protein (VP35), matrix protein (VP40), and RNA polymerase (L) ( Figure 1F). This suggests the presence of multiple lineages and not detection of reassortments due to the software sampling reads at multiple loci.

## Discussion

Detection of infectious disease pathogens is rapidly evolving to provide convenient point-of-need surveillance, ensuring rapid turn-around times for rational disease management. Third generation sequencing technologies, for example MinION nanopore sequencing are affordable, portable and popular in resource-poor endemic settings. Metagenomic data has the potential of being used as a routine clinical diagnostic tool for rapid detection of a wide range of infectious disease pathogens ^11, 34, 35^. Continuous discovery of novel pathogens, for example SARS-CoV-2 ^19^, MERS ^20^, and Bas-Congo rhabdovirus ^36^, highlights the need for broad-spectrum diagnostic tests capable of detecting divergent and emerging pathogens.

Computational analysis approaches for metagenomic data ought to be sensitive enough to detect pathogens in complex clinical samples open to the environment, such as those collected from stool, skin, and respiratory secretions. Multiple analyses strategies have been developed for virus sequence classification, but they have some limitations, for example the Virus Pathogen Resource (ViPR) is limited to only 14 of the 96 virus families ^16, 37^. Other tools only identify specific sequences, for example VirSorter and ClassyFlu for bacteriophage and influenza sequences respectively ^38, 39^. NOMAD not only robustly detects distant viral homologs compared to non-probabilistic alignment-based methods like BLAST and Bracken, but also classifies pathogens present in the universal virus taxonomy catalogue ^16, 40^. In addition, NOMAD is capable of circumventing challenges due to incomplete sequences, and sequencing and assembly errors, such as those arising in MinION nanopore sequencing, which pose a challenge to understanding pathogen transmission clusters and population structure ^41– 43^. Most existing tools are only able to correctly report the lowest common ancestor where multiple sequences share homology ^25^. NOMAD is able to distinguish between variants from the same species. Run time and memory use by these tools is determined by the disk speed of reading the reference sequence database into memory. Several detection tools have increased speeds at the expense of sensitivity, and their performance is expected to decrease with increase in reference database size due to redundancy ^31^. Although more computationally intensive because of the analysis of all six frames of potential DNA-to-amino acid translation, more advanced users can decrease NOMAD’s runtime by using customized databases of reduced sizes or parallel analysis using subsets of the entire database.

NOMAD can accurately distinguish between strains/lineages within viral species. It provides a more granular perspective of the composition of viral pathogens present in outbreak samples, which would otherwise require follow-up specialized analysis approaches for accurate strain typing ^31^. NOMAD was able to distinguish GC-poor phages, *Aeromonas phages - 25 and 44RR2*, which parasitize the GC-rich bacteria *Aeromonas salmonicida* ^44^, and identify phage *BcepNY3*, a new member of the *Burkholderia phage Bcep781* family that exhibit 87% – 99% sequence identity, whose host is soil bacteria of the *Burkholderia cepacia* complex ^45^. NOMAD also distinguished multiple strains of *Venezuelan equine encephalitis virus* (VEEV), a mosquito-borne positive-sense, single-stranded RNA *Alphavirus* that causes fatal encephalitis in human and equine hosts ^23^. This differentiation was also made between hepatitis C virus (HCV) genotype 1 subtypes 1a and 1b, which are largely responsible for treatment-resistant hepatic disease ^46^. There is close sequence similarity between all Ebola subtype Zaire viruses isolated from multiple geographic locations over the past 20 years, suggesting high conservation in their natural reservoir ^47^. Detection of multiple Ebola virus strains raised the possibility that there were sequencing artefacts or multiple linages of Zaire Ebola virus are present within the same intra-host disease episode. The detected Ebola virus strains are similar genetically, and previous phylogeny reconstruction showed they are in the same cluster, which is separate from those recently identified in Guinea, West Africa ^48, 49^. Virus evolutionary rate is higher during an outbreak and consists of numerous deleterious variants that are eventually eliminated by purifying selection, which is more relaxed in humans compared to the natural reservoir species ^47^. Presence of multiple variants was further confirmed by examination of reads from multiple locus, which included: non-structural protein (VP35), matrix protein (VP40), and RNA polymerase (L). We excluded glycoprotein (GP) that has the most pronounced genetic amino acid diversity ^47^. The diversity detected might be a result of sequencing artefacts, since the same MinION sequencer nanopore flow cell used to run the chikungunya sample was washed and re-used to sequence the Ebola virus dataset during the experiments ^11^. Overall, NOMAD has great sensitivity to detect and distinguish between viral lineages, and provides useful information on the composition of virus pathogens present in out-break isolates of NM-AFI. Etiology data on the most prevalent viral strains is insightful for the development of interventions, such as vaccines, and restricts probable vaccine escape by incorporating targets with broad coverage in the prevalent or emerging pathogens ^50^. The granular perspective on intra-host variants provided by NOMAD provides useful insights on transmission chains within communities, that facilitate targeted interventions to predominant and virulent pathogens in a particular endemic locale. Identificatio of intra-host variants has successfully been implemented to Ebola virus temporal and transmission patterns in the 2013 – 2016 outbreak to improve understanding in disease biology ^51^. 

## Conclusion

New findings on the viral composition of NM-AFI will enable improvements of case management and treatment outcomes, reduce antibiotic resistance due to presumptive prescriptions, strengthen outbreak detection and response, and improve the basis of treatment guidelines after understanding the viral landscape in endemic regions ^1, 2, 52^. Directed diagnostic tests, such as PCR, using specific viral biomarkers can then be developed from these new findings to improve routine disease monitoring in endemic resource-poor clinical settings.

## Data availability

### Underlying data

All data underlying the results are available as part of the article and no additional source data are required.

### Extended data

Zenodo: Reads of the iMESS simulated metagenomic datasets. https://doi.org/10.5281/zenodo.6558061 ^32^

- This project contains the following extended data: SM1.dataset1.25.01.21.Reads.txt
- SM2.dataset2.26.01.21.Reads.txt

Data are available under the terms of the Creative Commons Attribution 4.0 International license (CC-BY 4.0).

### Accession numbers

GenBank Sequence Read Archive: chikungunya virus Puerto Rico-S6 metagenome nanopore. SRP057410; https://identifiers.org/insdc.sra:SRP057410

GenBank Sequence Read Archive: Nanopore metagenome Ebola virus/H.sapiens-wt/COD/2014/Lomela-Lokolia16. SRP057409; https://identifiers.org/insdc.sra:SRP057409

GenBank Sequence Read Archive: Nanopore metagenome Ebola virus/H.sapiens-wt/COD/2014/Lomela-Lokolia11. SRX1023619; https://identifiers.org/insdc.sra:SRX1023619

GenBank Sequence Read Archive: HCV metagenome nanopore UCSF. SRX999819; https://identifiers.org/insdc.sra:SRX999819

GenBank Sequence Read Archive: HCV metagenome nanopore UCSF. SRX999819; https://identifiers.org/insdc.sra:SRX999819

GenBank Sequence Read Archive: REPLIg WTA of mosquito microbiome sequenced on ONT MinION. SRX3315008; https://identifiers.org/insdc.sra:SRX3315008

GenBank Sequence Read Archive: REPLIg WTA of mosquito microbiome sequenced on ONT MinION. SRX3315007; https://identifiers.org/insdc.sra:SRX3315007

GenBank Sequence Read Archive: SIGMA WTA of mosquito microbiome sequenced on ONT MinION. SRX3315006; https://identifiers.org/insdc.sra: SRX3315006

GenBank Sequence Read Archive: SIGMA WTA of mosquito microbiome sequenced on ONT MinION. SRX3315005; https://identifiers.org/insdc.sra:SRX3315005

## Software availability

Source code available from: https://github.com/kulohoma/NOMAD

Archived source code at time of publication: https://doi.org/10.5281/zenodo.6554784 ^21^.

License: CC0-1.0
