# Supplementary material for: NOMAD: metagenomic characterisation of the viral pathogen composition in outbreaks of non-malaria acute febrile illness cases
Source: Open Res Afr. 2025 Dec 1;5:24. Originally published 2022 Jun 9. [Version 2] doi: 10.12688/openresafrica.13406.2 (PMC12744201; doi:10.12688/openresafrica.13406.2)
Supplement: Peer review history

# Reviewer report: Reviewer response for version 2

**DOI:** 10.21956/openresafrica.17523.r33186

**Published:** 2025-12-27

**Authors:** Gerald Mboowa

**Affiliations:** 1 Broad Institute of MIT and Harvard, Cambridge, Massachusetts, USA

The authors have satisfactorily addressed all of my previous comments and concerns.

Are the conclusions about the tool and its performance adequately supported by the findings presented in the article?

Yes

Is the rationale for developing the new software tool clearly explained?

Yes

Is the description of the software tool technically sound?

Partly

Are sufficient details of the code, methods and analysis (if applicable) provided to allow replication of the software development and its use by others?

Partly

Is sufficient information provided to allow interpretation of the expected output datasets and any results generated using the tool?

Yes

Reviewer Expertise:

Pathogen genomics and bioinformatics (NGS) & antimicrobial resistance genomics,

I confirm that I have read this submission and believe that I have an appropriate level of expertise to confirm that it is of an acceptable scientific standard.

# Reviewer report: Reviewer response for version 1

**DOI:** 10.21956/openresafrica.14554.r32777

**Published:** 2025-11-11

**Authors:** Gerald Mboowa

**Affiliations:** 1 Broad Institute of MIT and Harvard, Cambridge, Massachusetts, USA

The manuscript does not describe the full NOMAD workflow end-to-end especially host/read decontamination, quality filtering, read-length/complexity filters, and how six-frame translation is integrated with HMMER. A schematic or stepwise section is needed so others can reproduce it.

 It is not stated whether human/mosquito reads were removed before HMM search. For clinical metagenomics this is critical; please specify reference genome used (e.g. GRCh38, *Aedes*), tool, and parameters.

 Abundance is said to be “determined by retention of duplicate reads,” but no formula, normalization (reads per million), or per-taxon counting rule is given. This must be formalized and justified.

 The paper does not specify what minimum number of reads, loci, or HMM hits is required to call a virus “present,” which makes the multi-virus calls (e.g. multiple Ebola lineages) hard to interpret. A sensitivity/specificity trade-off or ROC on the simulated data should be shown.

 The statistical comparison reports no significant difference, but the sample size is small and the biological interpretation of “NOMAD detects more lineages” is not quantified; add per-dataset precision/recall and runtimes for both methods.

 For the Ebola dataset, the authors note that the same MinION flowcell was re-used, so carryover could explain multiple strains; this should be framed more cautiously and supported with read-mapping depth by locus.

 The manuscript mentions ViPR, VirSorter, and ClassyFlu, but doesn’t show, even briefly, why NOMAD would be preferred for NM-AFI in African settings (e.g. ability to use MinION data with high error + broad viral DB + low compute). Add that comparison.

 Hardware requirements should be pinned so others can get the same results.

Are the conclusions about the tool and its performance adequately supported by the findings presented in the article?

Yes

Is the rationale for developing the new software tool clearly explained?

Yes

Is the description of the software tool technically sound?

Partly

Are sufficient details of the code, methods and analysis (if applicable) provided to allow replication of the software development and its use by others?

Partly

Is sufficient information provided to allow interpretation of the expected output datasets and any results generated using the tool?

Yes

Reviewer Expertise:

Pathogen genomics and bioinformatics (NGS) & antimicrobial resistance genomics,

I confirm that I have read this submission and believe that I have an appropriate level of expertise to confirm that it is of an acceptable scientific standard, however I have significant reservations, as outlined above.

# Response

**Published:** 2025-11-24

**Authors:** Benard Kulohoma

**Affiliations:** IAVI, Kenya

We thank **Reviewer 2** for the detailed and constructive comments aimed at improving the clarity and reproducibility of the manuscript. We address each point in turn and indicate the changes made to the revised manuscript, below:

**1. The manuscript does not describe the full NOMAD workflow end-to-end especially host/read decontamination, quality filtering, read-length/complexity filters, and how six-frame translation is integrated with HMMER. A schematic or stepwise section is needed so others can reproduce it.**

**Response: **We appreciate this suggestion. NOMAD is intentionally designed to be agnostic to upstream pre-processing steps because it accepts any pre-processed nucleotide FASTA sequences as input. Users may therefore apply their preferred quality filtering and host-read removal pipelines prior to running NOMAD; this flexibility is important in diverse field and laboratory settings. We also clearly share in the text that we used the EMBOSS suite for pre-processing: “The nucleotide query sequences in FASTA format were translated in all six reading frames using transeq in the EMBOSS software suite, prior to use as input data.” Moreover, a “read me” user guide is already provided on GitHub, which provides a full walkthrough of running NOMAD. To clarify this, we have revised the manuscript to explicitly state: “NOMAD assumes that upstream quality control and host-read removal are performed by the user, depending on sample type and experimental design.”

**2. It is not stated whether human/mosquito reads were removed before HMM search. For clinical metagenomics this is critical; please specify reference genome used (e.g. GRCh38, Aedes), tool, and parameters.**

**Response: **We clarify that NOMAD is **not** designed to classify mosquito or human genomic reads directly. Instead, NOMAD exclusively compares user-supplied sequences against viral protein HMMs constructed from **manually curated Swiss-Prot and TrEMBL viral proteins**. As such, host-read removal is assumed to be completed upstream by the user depending on the biological sample type. However, even in the event that this is not done, NOMAD matches only viral sequences in the HMMs constructed, and would therefore not match human or mosquito reads/sequences.

 In the manuscript we clarify that “Manually curated (n=563,630, as of 26th October 2020) and computer-annotated (n=1,200,724, as of 26th October 2020) viral protein sequences were retrieved from the Swiss-Prot and TrEMBL protein databases respectively, and used to construct profile HMMs.” The user can filter out human/mosquito or any other sequences not required for evaluation in the pre-processing steps.  

**3. Abundance is said to be “determined by retention of duplicate reads,” but no formula, normalization (reads per million), or per-taxon counting rule is given. This must be formalized and justified. The paper does not specify what minimum number of reads, loci, or HMM hits is required to call a virus “present,” which makes the multi-virus calls (e.g. multiple Ebola lineages) hard to interpret. A sensitivity/specificity trade-off or ROC on the simulated data should be shown.**

**Response: **We share that a stringent HMMER e-value threshold of < 1e-3 was applied to retain statistically significant hits. an e-value threshold of < 1e-3, a measure of the expected number of false positives given the size of the database, was applied to retain statistically significant hits. Only robustly identified sequences against the HMMs of the viruses of interest are counted to establish relative abundance. We have revised the manuscript to indicate that “Only sequences with robust HMM assignments (e-value <1e-3 and highest-scoring match to a viral family represented in the curated HMM set) are counted”. Relative abundance is based on the number of retained sequences matching each viral HMM, normalized by total retained hits per dataset. That is ≥1 significant HMM hit, confirmed across ≥1 locus for that viral HMM. Figure 1 demonstrates comparative sensitivity relative to Bracken, similar datasets.

**4. The statistical comparison reports no significant difference, but the sample size is small and the biological interpretation of “NOMAD detects more lineages” is not quantified; add per-dataset precision/recall and runtimes for both methods.**

**Response: **We clarified in the manuscript under the “metagenomics datasets and virus detection” section real word scenario datasets used to evaluate performance including relative abundance concordance with ground truth and comparative detection across tools. NOMAD consistently shows a slight performance edge (Figure 1) relative to Bracken for these datasets. Runtime has already been provided under the “Operation” section.

**5. For the Ebola dataset, the authors note that the same MinION flowcell was re-used, so carryover could explain multiple strains; this should be framed more cautiously and supported with read-mapping depth by locus.**

**Response: **We share that "Presence of multiple variants was further confirmed by examination of reads from multiple locus, which included: non-structural protein (VP35), matrix protein (VP40), and RNA polymerase (L). We excluded glycoprotein (GP) that has the most pronounced genetic amino acid diversity. The diversity detected might be a result of sequencing artefacts, since the same MinION sequencer nanopore flow cell used to run the chikungunya sample was washed and re-used to sequence the Ebola virus dataset during the experiments." We are cautious about our findings and try to highlight some of the reasons, from previously analysed work why considering multiple loci is necessary. We acknowledge that sequencing carryover or artefacts may partially explain the observed diversity.  

**6. The manuscript mentions ViPR, VirSorter, and ClassyFlu, but doesn’t show, even briefly, why NOMAD would be preferred for NM-AFI in African settings (e.g. ability to use MinION data with high error + broad viral DB + low compute).**  

**Response: **NOMAD has the ability to handle high-error MinION reads following six-frame translation and  accurately distinguish between strains/lineages within viral species.  It provides a more granular perspective of the composition of viral pathogens present in outbreak samples, which would otherwise require follow-up specialized analysis approaches for accurate strain typing. It is suitable for resource-poor environments through efficient HMM-based searches. The other classifiers listed by the reviewer had limitations. We have shared this is the manuscript text:  "Multiple analyses strategies have been developed for virus sequence classification, but they have some limitations, for example the Virus Pathogen Resource (ViPR) is limited to only 14 of the 96 virus families16,37. Other tools only identify specific sequences, for example VirSorter and ClassyFlu for bacteriophage and influenza sequences respectively".   “Overall, NOMAD has great sensitivity to detect and distinguish between viral lineages, and provides useful information on the composition of virus pathogens present in out-break isolates of NM-AFI. Etiology data on the most prevalent viral strains is insightful for the development of interventions, such as vaccines, and restricts probable vaccine escape by incorporating targets with broad coverage in the prevalent or emerging pathogens. The granular perspective on intra-host variants provided by NOMAD provides useful insights on transmission chains within communities, that facilitate targeted interventions to predominant and virulent pathogens in a particular endemic locale”.  

**7. Hardware requirements should be pinned so others can get the same results**.

**Response: **We shared the hardware used for comparison, under the “Operation” section, see below:

 " **Operation** The average analysis runtime for ~50,000 metagenomics reads was 60 minutes. This analysis was conducted on our distributed HPC cluster, with an Ubuntu 20.04.3 LTS operating system, with each node being an Intel(R) Xeon(R) Silver 4214 CPU @ 2.20GHz with 12 cores". This will enable reproducibility by other users.   We thank the reviewer for the insightful feedback that has significantly strengthened the manuscript. The manuscript has been revised to improve clarity and methodological transparency.

# Reviewer report: Reviewer response for version 1

**DOI:** 10.21956/openresafrica.14554.r30054

**Published:** 2023-08-11

**Authors:** Sophana Chea

**Affiliations:** 1 International Center of Excellence in Research, National Institutes of Health, Phnom Penh, Cambodia

**Summary**

 Metagenomic sequencing is important to determine the unknown sample, especially to predict the outbreak of novel pathogens. The sequencing technique relies on precise laboratory procedures and needs a powerful data analysis platform. NOMAD (Non-Malaria Acute febrile illness Detector) is a platform specialized in detecting multiple viral pathogens in sequencing data. In this paper, NOMAD was performed to classify sequences in the taxa and estimate viral species compared to an analysis by Bracken (Bayesian Re-estimation of Abundance after Classification with KrakEN). Several metagenomic datasets were used in the analysis, such as GenBank’s Sequence Read Archive (SRA), the metagenomic dataset from MinION nanopore such as chikungunya virus, Ebola virus, Hepatitis C virus, and mosquito microbiome.

**Question**

 The authors clearly explained the rationale for developing the new software tool due to the limitation of the low-frequency sequence in the sequencing data is challenging. Some sequencing analysis tools require the full genome of the pathogen or contig sequence to get a confident identity result. NOMAD tool provides a range of technical pipeline use such as Hidden Markov Models (HMMs) and MUSCLE alignment tool.

1. In Response to the analysis method, Could authors provide more detail on the pipeline NOMAD uses? 
2. How does the NOMAD remove the host sequence in the sequencing data?
3. How does the NOMAD tool identify the abundance of sequencing in the dataset? 
4. What threshold number of reads provides confidence to call the pathogen in the sample?

Are the conclusions about the tool and its performance adequately supported by the findings presented in the article?

Yes

Is the rationale for developing the new software tool clearly explained?

Yes

Is the description of the software tool technically sound?

Yes

Are sufficient details of the code, methods and analysis (if applicable) provided to allow replication of the software development and its use by others?

Partly

Is sufficient information provided to allow interpretation of the expected output datasets and any results generated using the tool?

Partly

Reviewer Expertise:

Molecular sequencing

I confirm that I have read this submission and believe that I have an appropriate level of expertise to confirm that it is of an acceptable scientific standard.
